# Born high, born fast: Does highland birth confer a pulmonary advantage for sea level endurance?

**DOI:** 10.1113/EP091830

**Published:** 2024-11-22

**Authors:** Hunter L. Paris, Marissa N. Baranauskas, Keren Constantini, Ren‐Jay Shei, Peyton E. Allen, John R. Jadovitz, Chad C. Wiggins, Cooker Perkins Storm

**Affiliations:** ^1^ Division of Natural Sciences Pepperdine University Malibu California USA; ^2^ Department of Human Physiology & Nutrition University of Colorado Colorado Springs Colorado USA; ^3^ Sylvan Adams Sports Institute, Faculty of Medical & Health Sciences Tel Aviv University Tel Aviv Israel; ^4^ Indiana University Alumni Association Bloomington Indiana USA; ^5^ Department of Kinesiology Michigan State University East Lansing Michigan USA

**Keywords:** hypoxia, high altitude, performance, sea level

## Abstract

Less than 7% of the world's population live at an altitude above 1500 m. Yet, as many as 67% of medalists in the 2020 men's and women's Olympic marathon, and 100% of medalists in the 2020 men's and women's Olympic 5000 m track race may have been born or raised above this otherwise rare threshold. As a possible explanation, research spanning nearly a quarter of a century demonstrates that indigenous highlanders exhibit pulmonary adaptations distinct from their lowland counterparts. These adaptations may then promote endurance performance. Indeed, healthy indigenous highlanders often exhibit a larger aerobic exercise capacity compared to sea‐level residents who travel to high altitude. However, questions remain on whether high‐altitude birth is advantageous for sea‐level competitions. In this review, we ask whether being born at a high altitude generates an ergogenic advantage for endurance performance in the Summer Olympics—a venue that is generally held at sea level. In so doing, we distinguish between three groups of high‐altitude residents: (i) the indigenous highlander, (ii) the highland newcomer, and (iii) the highland sojourner. Concentrating specifically on altitude‐induced alterations to pulmonary physiology beginning in the perinatal period, we propose that if altitude‐related maladaptations are avoided, genomic and developmental alterations accompanying highland birth may present benefits for endurance competitions at sea level.

## Abstract (highlights)

- **What is the topic of this review?**
  Whether being born at high altitude contributes to endurance exercise performance at sea level. Does highland birth set the stage for lowland success?
- **What advances does it highlight?**
  The physiological effects and ergogenic potential of highland birth, especially as it pertains to the respiratory system. Possible (mal)adaptations evoked by perinatal hypoxia in various groups of highlanders and the influence of these pulmonary‐related alterations on endurance performance at sea level, for example, in the Summer Olympics.

## 1 INTRODUCTION

Mexico City, 1968. The race—or rather the outcome—is well known in the annals of Olympic Track & Field. Unbeaten in 3 years and with over 47 consecutive victories in the 1500 m and mile, Jim Ryun of the USA stood as the heavy favourite to win the 1500 m final. Contrary to expectations, however, it was Kip Keino of Kenya who broke the victor's tape that day in a time of 3:34.9. In attempting to explain this unexpected result, individuals often note the high altitude of Mexico City (2250 m) and point toward the Ryun versus Keino duel as evidence of the deleterious effects of altitude on endurance performance. Pundits also use the 1968 Olympics as a supportive example of the ergogenic effects of altitude acclimatization prior to racing at these higher elevations (Chapman et al., 2013). We do well to remember, however, that Jim Ryun did engage in altitude training in advance of the Mexico City Olympics and yet still lost to Keino by nearly 3 s. Although acknowledging the mental and sociological demands of any race‐day performance, Keino's defeat of Ryun permits additional inquiry into the nature and nuance of high altitude exposure on endurance capacity. Given that Ryun was born in Wichita, Kansas (∼400 m) and Keino in the Nandi Hills of western Kenya (∼2000 m), another consideration is whether being *born* at high altitude confers advantages for endurance performance.

**FIGURE 1:**
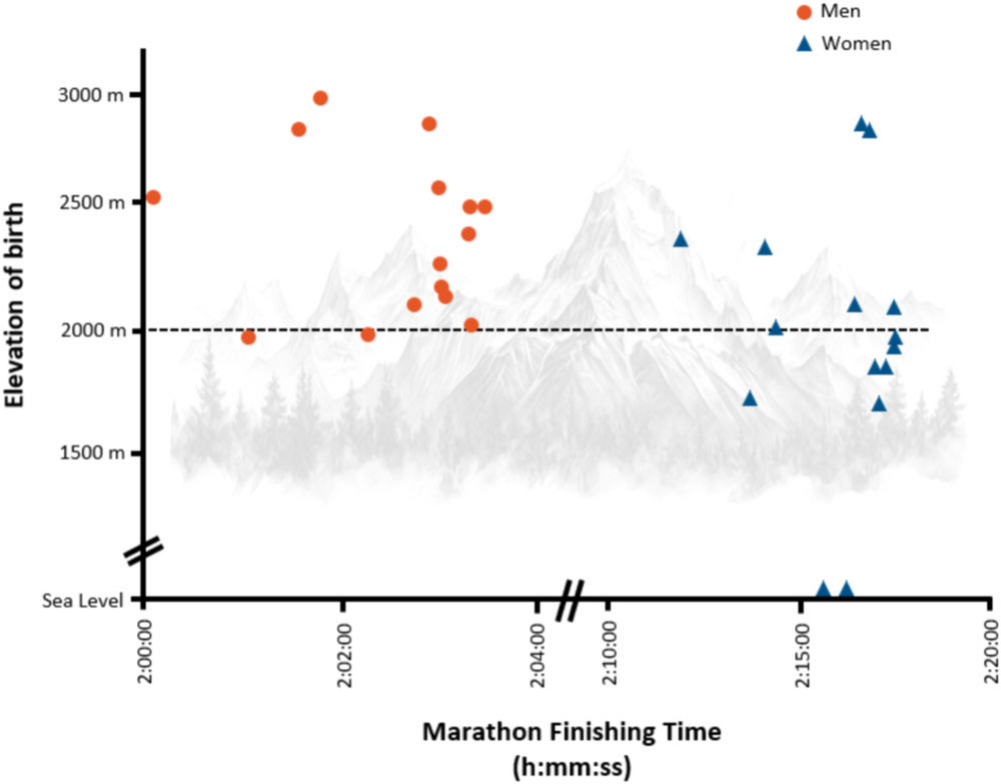
Approximate elevation of birth for the 20 fastest marathon men and 20 fastest marathon women of all time. (Data obtained from publicly available internet sources and current as of March 2024; unable to identify the town of birth for 5 men and 5 women.)

For competitions occurring at high altitude, endurance capacity is thought—though not without debate—to be greater in highland natives compared to those born at sea level (Lundby & Calbet, 2016; Wu & Kayser, 2006). The proficiency of highlanders to attenuate the decline in oxygen delivery when subject to low oxygen environments becomes particularly prominent when envisioning the performance of the Sherpas ascending the world's highest peaks. These physiological distinctions between native highlanders and lowlanders remain evident even when lowlanders acclimatize to high altitude, perhaps indicating inherited or developmental adaptations that differ from, and are predominant over, strategies of transient acclimatization (Brutsaert, 2008). But aside from the well‐known 1968 Olympics in Mexico City, Summer Olympics are consistently held at sea‐level venues and questions diminish regarding the possible aerobic merits of highland birth. However, since aerobic capacity is limited by oxygen handling in both high‐altitude and low‐altitude settings, physiological adjustments that optimize oxygen flux potentially benefit endurance performance in both environments (Wagner, 1996). This is one reason why stints of altitude training may be leveraged for sea‐level performance (Stray‐Gundersen et al., 2001). If adaptations accompanying highland birth differ from the acclimatization accompanying highland exposure, then performance outcomes—even at sea level—may likewise differ between these groups. A cursory look at performance results coupling elevation of birth with record‐breaking marathon performance supports this line of inquiry (Figure 1). Indeed, the physiological perturbations accompanying highland birth are distinct from adult high‐altitude acclimatization (Mairbäurl et al., 2020).

**FIGURE 2:**
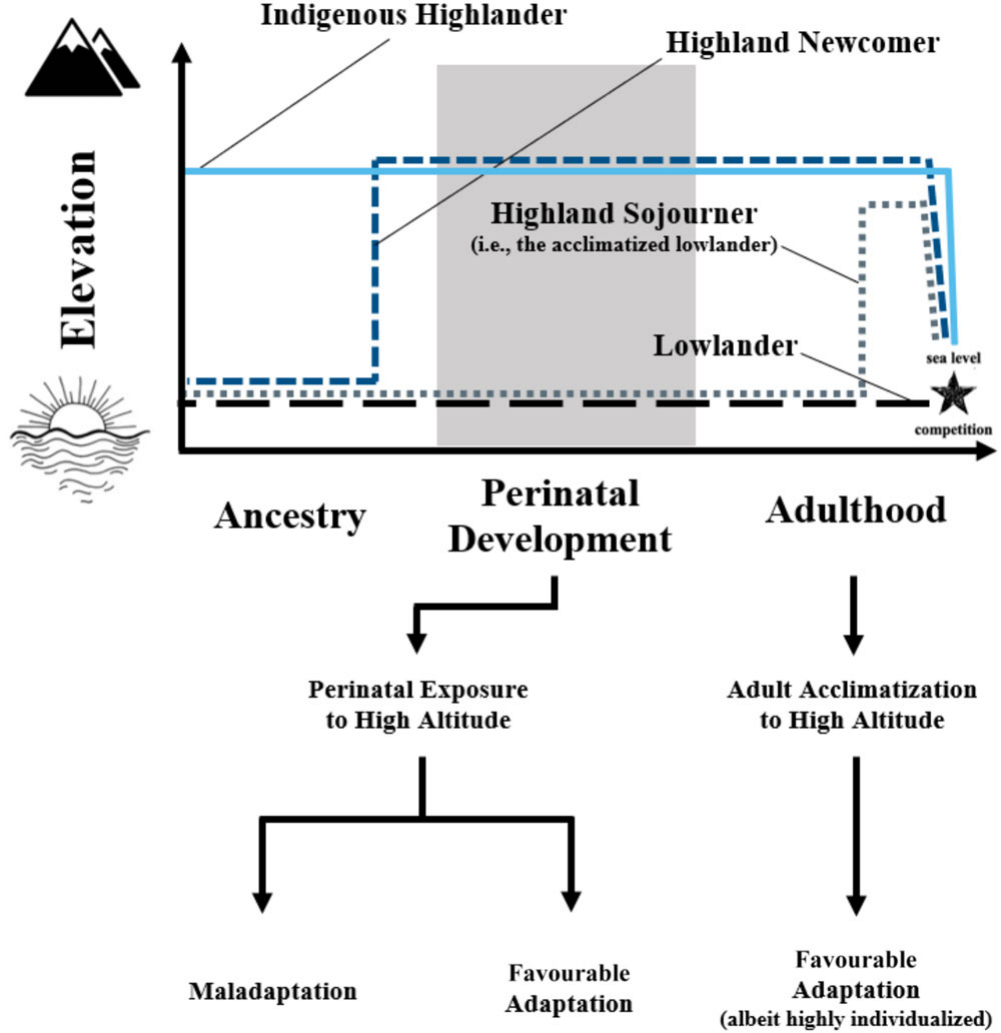
Schematic representation of hypothesis relating various exposure times to high altitude and their proposed influence on endurance performance at sea level (indicated by the star). For the indigenous highlander, high altitude exposure in the perinatal period occurs following generations of high altitude ancestry. The highland newcomer represents perinatal exposure but without the added adaptations from generational predecessors. Perinatal exposure to high altitude (be it indigenous highlander or newcomer) could result in increased ergogenic potential for endurance performance at sea level, but might also result in maladaptation. Exposure to high altitude as an adult (highland sojourner) induces an acclimatization response that is individualized and potentially ergogenic.

Given that the pulmonary system exhibits a high degree of developmental plasticity in response to environmental cues (Carroll, 2003), ventilatory control appears to be particularly susceptible to hypoxia during critical points of fetal development. Although the consequences of these alterations have largely been scrutinized for their role in the innate exercise capacity at high altitude, a lack of evidence dictates that questions remain regarding whether these differences affect endurance performance at sea level. In this review, and with a concentration on the Summer Olympics, we apply the documented physiological differences of various time‐course exposures to high altitude—particularly in altitude‐induced remodelling of the respiratory system—to endurance performance at sea level, asking whether highland birth facilitates lowland endurance. We direct this application towards endurance events that require a sustained aerobic effort at a near‐maximal intensity. For example, in Olympic running events this includes distances from 1500 m (performed near 100% of ${\dot{V}}_{O_{2}\max}$) up to the marathon (performed by elite‐level women and men at approximately 90% of ${\dot{V}}_{O_{2}\max}$) (Billat et al., 2001; Molinari et al., 2020). We hypothesize that, given an otherwise healthy pregnancy, highland‐born individuals combine genomic and developmental pulmonary adaptations in a way that confers an ergogenic advantage for sea‐level competition (Figure 2).

## 2 PULMONARY DEVELOPMENT GIVEN PERINATAL HYPOXIA

Perinatal exposure to high altitude imparts various anatomical and physiological responses in offspring. If these responses impair health and are detrimental to performance, then highland birth is not to be espoused for any ergogenic potential. Alternatively, if offspring responses to high altitude birth are meritorious and garner a more favourable outcome than altitude training as an adult, why not begin altitude exposure—some may wonder—during pregnancy? Addressing this requires a brief review of the maternofetal response to hypoxia, especially as it pertains to the respiratory system.

**FIGURE 3:**
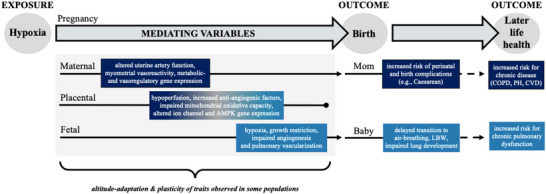
Pregnancy at high altitude. Hypoxia‐related challenges to maternal and fetal outcomes and the physiological variables that provoke these outcomes. COPD, chronic obstructive pulmonary disease; CVD, cardiovascular disease; LBW, low birth weight; PH, pulmonary hypoxia.

For native lowlanders experiencing pregnancy at high altitude, hypoxia can elicit harmful effects (outlined in Figure 3). As adequate oxygenation is crucial for tissue development and survival, chronic exposure to high altitude during pregnancy challenges the healthy development and function of various maternal, placental, fetal and newborn traits that determine pulmonary characteristics. More specifically, the acute impact of high altitude on pregnancy includes, but is not limited to: (1) maternal: lower uterine artery blood flow, altered myometrial vasoreactivity, and altered metabolic and vasoregulatory gene expression; (2) placental: hypoperfusion, increased anti‐angiogenic factors, impaired mitochondrial oxidative capacity, altered ion channel gene expression and insufficient AMP‐activated protein kinase expression; (3) fetal: hypoxia, growth restriction, impaired angiogenesis, and pulmonary vascularization; and (4) newborn: low birth weight, delayed or incomplete cardiopulmonary transition, and alveolar simplification (Heath‐Freudenthal et al., 2024; Wilsterman & Cheviron, 2021).

The interruption of the complex perinatal sequelae owing to hypoxia is contingent upon preserving oxygen delivery to the tissues. Acquired adaptations from generational exposure to high altitude may preserve oxygen delivery, mitigating some of the detrimental responses. One example of an acquired maternal adaptation is seen in the differences in uterine artery function between highlanders and lowlanders, such that the altitude‐associated reduction in uterine artery diameter and blood flow are attenuated in many who are indigenous to high altitude compared with native lowlanders whose pregnancy occurs at high altitude (Moore, 2021). The feto‐protective advantages of ancestral highlanders may best be observed in differences in birth weight—the single most important predictor of childhood morbidity and mortality. A consistent inverse dose‐dependent relationship exists between altitude and birth weight, demonstrating a 97 g birth weight reduction for every 1000 m of elevation gain (Yang et al., 2020). Although the impact of fetal growth restriction is associated with co‐morbidities at birth, there is also a well‐known relationship between low birth weight and later‐life disease. This relationship, known as the ‘Barker Hypothesis’ or ‘Fetal Origins of Adult Disease’, has spurred numerous studies that demonstrate a significant correlation between low birth weight and pulmonary impairment as an adult. Perinatal hypoxia's aforementioned effects on the pulmonary system (e.g., impaired pulmonary vascularization, delayed cardiopulmonary transition and alveolar simplification), with or without fetal growth restriction, lead to deleterious fetal outcomes that prevail across the lifespan. And yet again, some groups of indigenous highlanders appear to have garnered sufficient feto‐protective altitude adaptations where these babies are born without growth restriction (Bennett et al., 2008). In these indigenous highlanders especially, it is possible that genetic and developmental adaptations limit hypoxia‐related impairments in pulmonary function and, perhaps, even promote hypoxia‐related improvements.

It is clear that pregnancies can suffer egregiously in high‐altitude environments where oxygen‐preserving techniques lead to maladaptive responses that result in deleterious fetal outcomes, irreversible morbidities, or worse, infant mortality—a fate experienced by the first generations of lowland migrants to highland environments (Heath‐Freudenthal et al., 2024). Over time, those with longstanding highland ancestry may demonstrate maternal, placental and fetal approaches that mitigate the otherwise typical negative altitude‐induced responses, and offspring of these ancestral highlanders may gain anatomical and physiological adaptations that promote surviving—and possibly thriving—at high altitude. Whether these observed refinements in pulmonary function prove advantageous for endurance performance depends, in part, on whether pulmonary function limits endurance performance. It is generally agreed that pulmonary characteristics influence endurance competitions occurring at high altitude (Wagner, 1996). Whether pulmonary characteristics limit sea‐level performance is less apparent and warrants additional consideration.

## 3 DO THE LUNGS INFLUENCE SEA LEVEL PERFORMANCE?

For aerobic exercise at sea level, performance limitations are commonly associated with inadequate cardiac output or sub‐optimal haemoglobin concentration, and less so with any limitation imposed by the lungs or respiratory muscles (Wagner, 1996). During aerobic exercise, the respiratory system acts to replenish O_2_, relinquish CO_2_ and regulate pH. Even when exercise intensity is severe and duration is prolonged, the respiratory system of the healthy, recreationally trained individual sufficiently responds to increases in metabolic demand and maintains arterial oxygenation near resting levels (Peters et al., 2023). However, given that training‐induced remodelling of the lungs is limited (Peters et al., 2023; Wagner, 2005), the relatively robust training‐induced remodelling of the heart, blood and muscles may evoke a demand for oxygen that outpaces the ability of the lungs and respiratory muscles to meet this demand (Dempsey, 1986). Thus, the anatomical and physiological plasticity of the cardiovascular and muscular systems underlies a gap in symmorphosis where the capacity of the respiratory system lags behind, thereby potentially generating pulmonary constraints that may hinder aerobic performance. For example, as ventilation intensifies so too does the effort of the respiratory muscles and thus the oxygen cost of breathing (Babcock et al., 2002). The effects of high altitude on the work of breathing lack clarity however, with conflicting reports on whether high altitude increases or decreases the work of breathing (Cibella et al., 1999; Petit et al., 1963; Thoden et al., 1969). Minute ventilation rate (*V̇*_E_) at any given work rate is typically higher at high altitude compared to sea level, and despite lower air density, it is unclear whether the lower air density contributes to a lower airway resistance, due to multiple physiological responses to high altitude. Although lower air density, absent any other physiological changes, would indeed reduce airway resistance, at high altitude a combination of factors such as hypoxia, hypocapnia, altered breathing patters and operating lung volumes, vascular, and endocrine factors may all affect airway resistance, and in some cases, increase airway resistance in spite of lower air density (Cibella et al., 1999). Increased airway resistance in these instances may be due to bronchoconstriction or pulmonary oedema. Additionally, lung hyperinflation (i.e., an increase in the end‐expiratory lung volume) may also increase the work of breathing. Corresponding with increased respiratory muscle work is an evoked sympathetic response that constricts blood flow to exercising limbs. Although this respiratory muscle metaboreflex preserves blood flow to the respiratory muscles, it compromises oxygen delivery to locomotor muscles and represents a potential pulmonary constraint on exercise performance (Amann et al., 2007). Pulmonary limitations exist beyond the work of breathing, and other examples where capabilities of the lungs and respiratory muscles confine aerobic capacity include expiratory flow limitations, impaired gas exchange and exercise‐induced arterial hypoxaemia, and inadequate pulmonary capillary blood volume (Peters et al., 2023). Ultimately, do pulmonary characteristics *influence* endurance capacity at sea level? Yes (Amann, 2012). Do pulmonary characteristics *limit* endurance capacity at sea level? It depends. Although the upper bounds of the respiratory system are often sufficient to meet the metabolic demands of exercise, pulmonary limitations may become particularly prominent in Olympic‐calibre athletes because of the very high demand for oxygen transport to the working muscle (Dempsey et al., 2020). Therefore, if a high altitude birth engenders a high functioning pulmonary network, it holds potential to influence performance outcomes, even at sea level.

## 4 HIGHLAND POPULATIONS

### 4.1 The highlander, the newcomer and the sojourner

As used by biological anthropologists, the term *acclimatization* refers to short‐term (hours to weeks) physiological changes elicited by a novel environment, whereas *adaptation* refers to long‐term alterations in anatomy, physiology and the developmental processes that occur over generations (Niclou et al., 2023). Even within these definitions, subtlety exists, where the physiological alterations resulting from perinatal exposure to high altitude depend upon the degree of highland ancestry. Therefore, even when acknowledging the likelihood of disparate physiologies between the highland‐born and lowland‐born, dose‐dependent responses exist and merit consideration for the generations of highland ancestry within the highland‐born.

In acknowledging the potential inheritability of adaptations to high altitude we distinguish between three populations experiencing altitude‐induced physiological remodelling: indigenous highlanders, highland newcomers and highland sojourners. *Indigenous highlanders* are those native to high altitude with multigenerational exposure to high altitude. Aligning with those studies investigating the biological adaptations to altitude, indigenous highlanders are defined as those who were born and raised above 2500 m, and whose ancestors were likewise (Beall, 2007; Moore et al., 1998). Those that fall into this category include those of the Andean mountains (Quechua and Aymara), the Tibetan Plateau (Tibetans and Sherpa) and the Ethiopian highlands (Amhara). *Highland newcomers* include people born and/or raised from early adolescence at high altitude but who lack long‐term generational exposure. Examples of highland newcomers above 2500 m include those from Leadville, Colorado as well as non‐indigenous individuals of La Paz, Bolivia. Within this group of highland newcomers, perhaps the most studied is the Han (Chinese) migrants to the Tibetan plateau—a migration of lowlanders to the highland environs of the Himalayan mountains that occurred en masse in the early 1950s (Weitz et al., 2013). Finally, *highland sojourners* are individuals born and raised near sea level who ascend and acclimatize to high altitude following adolescent development. The common scenario in this category includes sea‐level athletes journeying to high altitude for stints of altitude training, though this would be achieved at elevations closer to 2000 m. As such, while a consensus on what constitutes ‘high altitude’ is difficult to procure, in this review we refer to high altitude as any elevation ≥2000 m unless indicated otherwise.

## 5 INDIGENOUS HIGHLANDERS

### 5.1 Pulmonary adaptations in those with highland ancestry

**FIGURE 4:**
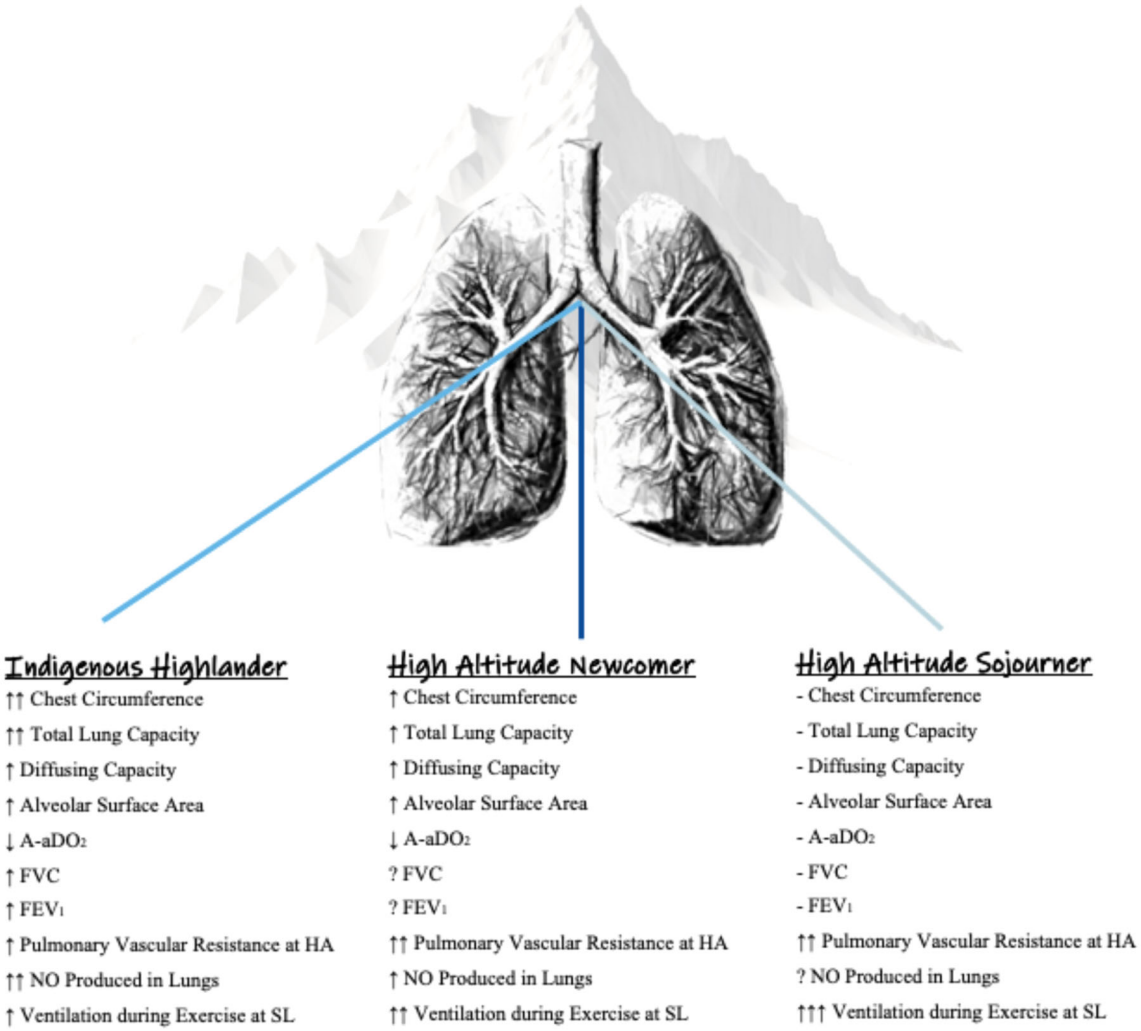
Likely pulmonary anatomical and physiological distinctions between highland populations compared with lifelong sea level residents. Reported characteristics are those which are observed in highland natives, newcomers and sojourners upon acute exposure to sea level. $A-aD_{O_{2}}$, alveolar‐arterial $P_{O_{2}}$ difference; FEV_1_, forced expiratory volume in 1 s; FVC, forced vital capacity; HA, high altitude; NO, nitric oxide; SL, sea level.

Indigenous highlanders exhibit increased chest circumferences, tidal volumes, residual volumes and vital capacities (and therefore total lung capacities) compared with lowlanders (Brutsaert et al., 1999; Gilbert‐Kawai et al., 2014) (Figure 4). Greater lung volumes are accompanied by altered lung mechanics, and some evidence suggests that highland natives demonstrate larger values in forced vital capacity (FVC), forced expiratory volume in 1 s (FEV_1_), and peak expiratory flow rates compared to lowlanders (Havryk et al., 2002; Wood et al., 2003). Highland birth and ancestry are also accompanied by relative hypoventilation at rest in hypoxia, particularly in Andean natives (Brutsaert, 2007). Greater lung volumes correspond with increases in alveolar surface area and capillary blood volume, which may result in larger pulmonary diffusing capacity (measured, for example, as the diffusing capacity for carbon monoxide; DLCO) (Jones et al., 1992) and an accompanying ability to defend arterial oxygen saturation ($S_{aO_{2}}$) in hypoxia. A 2007 review comparing indigenous highlanders and newcomers found no significant difference in $S_{aO_{2}}$ at rest but indicated that indigenous highlanders better preserve $S_{aO_{2}}$ during conditions of hypoxaemic stress such as exercise (Weitz & Garruto, 2007). To this end, when performing cycling exercises in hypobaric hypoxia, Tibetan natives recorded higher $S_{aO_{2}}$ values compared to their Han counterparts (Ge et al., 1995). Though evidence conflicts regarding differences in $S_{aO_{2}}$ between highlanders and lowlanders (Gilbert‐Kawai et al., 2014; Wu & Kayser, 2006), data point toward a greater diffusing capacity in highland natives, and also toward a smaller alveolar–arterial $P_{O_{2}}$ difference ($A-aD_{O_{2}}$), even at sea level (Julian & Moore, 2019). For example, non‐native residents of high altitude exhibit higher DLCO at rest and during exercise compared with lowland residents, regardless of whether highlanders began altitude residence in adulthood or adolescence (Cerny et al., 1973). However, the younger resident group (which settled in high altitude during adolescence before physiological maturity) more closely resembled the characteristics of native highlanders in the study. The increased diffusing capacity in these high‐altitude residents was found not to be due to differences in haemoglobin concentration or alveolar lung volume, but rather the biggest contributing factor was an increase in diffusing membrane capacity (Cerny et al., 1973).

### 5.2 Implications for performance at sea level

If Dempsey's classic hypothesis rings true—that the lungs do in fact represent a limiting factor in endurance‐trained athletes *even at sea level* (Dempsey et al., 1984)—then the physiological merits of highland ancestry may confer an advantage at sea level. Additionally, although inadequate hyperventilation compromises $S_{aO_{2}}$, the documented low ventilation of the indigenous highlander (compared to the ventilations of the newcomer and sojourner) may prevent blood from being shunted toward respiratory muscles and so better preserve oxygen delivery to exercising limbs. By mitigating the work of breathing then, the low ventilatory response of the indigenous highlander may lessen the respiratory muscle metaboreflex (compared to the newcomer or sojourner) and more effectively sustain heavy‐intensity exercise (Constantini, Tanner et al., 2017; Harms et al., 2000). Exercising ventilation alone is insufficient to confirm this possibility, but measures of skeletal muscle oxygen and neuromuscular fatigue during exercise would be helpful toward this end. If the metaboreflex was not involved, then reduced ventilation may in fact impair performance by compromising oxygen saturation if the ventilation is insufficient to meet oxygen demand. Ultimately, whereas an increased ventilation may preserve arterial oxygenation thereby lessening fatigue (Amann et al., 2007), it may alternatively elevate the work of breathing thereby magnifying fatigue (Amann et al., 2007). In the absence of direct experimentation, the ventilatory response for any of the various highland populations (be they indigenous highlanders, newcomers or sojourners) is difficult to assess for their performance implications at sea level.

## 6 HIGHLAND NEWCOMERS

### 6.1 Pulmonary adjustments in those with perinatal—but not ancestral—highland exposure

In the early 1970s, Roberto Frisancho found that lowlanders raised in the Andean highlands since childhood exhibited pulmonary function and aerobic capacities (at high altitude) similar to indigenous highlanders, and concluded that developmental exposure to high altitude expands lung volume and promotes alveolar gas exchange (Frisancho, Martinez et al., 1973; Frisancho, Velásquez et al., 1973). These observations point toward altitude‐induced adaptations in pulmonary form and function when individuals are exposed to high altitude during childhood—with or without highland birth and ancestral heritage. This is understandable when remembering that the alveolar phase of lung development continues for many years post‐natal. Animal models support Frisancho's conclusions, showing that newborn rats reared in high altitude environments develop alveolar proliferation, larger lung volumes, and a larger diffusing capacity compared to sea level controls (Burri & Weibel, 1971). Increased lung volumes are likely the case for highland newcomers as well, although when highland newcomers are compared to those with generational exposure to high altitude, the indigenous highlanders exhibit lung volumes larger still (Figure 4) (Brutsaert et al., 1999; Weitz et al., 2013). Perinatal exposure to high altitude also increases vital capacity (in both natives and newcomers) compared to lowlanders (Weitz et al., 2013). Canine studies in which animals were subjected to 5 months of high altitude residence during maturation indicate that developmental adaptations of the lungs, especially enhancement in pulmonary diffusing capacity ($D_{LO_{2}}$, measured using the multiple inert gas elimination technique), were preserved at sea level, and persisted for up to 2 years after the return to sea level (Hsia et al., 2007). Regarding hypoxia‐induced increases in ventilation, the ventilatory response to hypoxia is a function of the length of exposure, and newcomers exhibit a dampened chemosensitivity and ventilatory response to hypoxia *compared with highland sojourners* (Weil et al., 1971). Overall findings therefore suggest that, similar to indigenous highlanders, highland newcomers exhibit increases in lung volume and enhanced diffusing capacities that are retained upon return to sea level.

### 6.2 Implications for performance at sea level

Comparing maximal aerobic capacity (${\dot{V}}_{O_{2}\max}$) values *at high altitude*, ${\dot{V}}_{O_{2}\max}$ is 8% higher in highland newcomers compared to acclimatized sojourners thus indicating some degree of anatomical or physiological difference between the two groups. Yet, indigenous highlanders exhibit ${\dot{V}}_{O_{2}\max}$ values that are *an additional* 8% higher (therefore 16% above the acclimatized sojourner) (Moore et al., 1998). These values pertain to ${\dot{V}}_{O_{2}\max}$ at high altitude, and while the differences between groups weaken when descending to sea level, the observed differences at high altitude may exemplify a gradation of ergogenic enhancement conferred by the length of hypoxic exposure (Moore et al., 1998). Although it remains speculative, improvements in the diffusion capacity of the lung for oxygen may confer an ergogenic advantage at sea level. Evidence also suggests a smaller $A-aD_{O_{2}}$ in the newcomer compared to the sojourner, but it is unclear whether $S_{aO_{2}}$ is better preserved in the newcomer (Dempsey et al., 1971; Wagner et al., 2002). Regarding the ventilatory response to hypoxia, Rocky Mountain highlanders (e.g., those from Leadville, Colorado with less than 200 years of highland ancestry) exhibit lower ventilation at high altitude than acclimatized sojourners (Beall et al., 1997; Weil et al., 1971). Similar to indigenous highlanders then, if reduced chemosensitivity lessens exercise ventilation at sea level (compared to highland sojourners), this may reduce respiratory muscle work, preserve blood flow to exercising muscles, and improve sea level performance (Harms et al., 2000).

## 7 HIGHLAND SOJOURNERS

### 7.1 Pulmonary acclimatization in lowlanders who travel to altitude as adults

For the lowland adult athlete ascending to a high‐altitude training camp, reductions in the partial pressure of oxygen ($P_{O_{2}}$) stimulate sympathetic activity and pulmonary ventilation within minutes (Calbet & Lundby, 2009; Hoiland et al., 2018). Upon return to sea level, ventilation remains elevated above pre‐altitude ventilation for at least 1–2 days after arrival (Chapman, Karlsen et al., 2014; Levine & Stray‐Gundersen, 1997; Wilhite et al., 2013) due to the sudden cessation of the hypoxic ventilatory response, resulting in a relatively acidic pH stimulating the chemoreceptors.

### 7.2 Implications for performance at sea level

In collating data to provide recommendations for optimizing stints of high altitude training for sea level residents, Constantini and colleagues (Chapman, Laymon et al., 2014; Constantini, Wilhite 2017) cite three primary concerns regarding when athletes should return to sea level. Two of these considerations pertain to breathing patterns altered by the hypoxic environment that persist upon initial return to normoxia. The first concern is that exercising ventilation remains transiently elevated upon sea level exposure. Increases in ventilation incite increases in the perception of breathlessness (dyspnea) and may hinder endurance performance due to an increased metabolic cost of breathing (Wilhite et al., 2013). The second concern pertains to biomechanical alterations accompanying the highland environment. One aspect of this involves locomotor–respiratory coupling—the conjoining of ventilation with locomotion where breaths begin at specific time points within each stride (Fulton et al., 2018). Similar to the hyperventilatory response, hypoxia‐induced changes to locomotor–respiratory coupling may increase the oxygen cost of breathing and worsen exercise tolerance (Hoffmann et al., 2012). The aforementioned concerns are expected to subside given enough time (perhaps up to 2 weeks) back at sea level (Constantini, Wilhite et al., 2017).

## 8 POTENTIAL MALADAPTATIONS

In 1948, Carlos Monge wrote, ‘It is to be inferred that the individual living at high altitude either becomes an athlete, as it were, or perishes as a victim of fatigue’ (Monge, 1948). Although the present review considers high altitude in light of its potential physiological merits, hypoxia is often viewed as anathema to physiological health and fitness. Hypoxia presents an offense to tissue oxygenation, and even the adaptations experienced by the indigenous highlander may be insufficient to completely abolish the stress of hypoxia. Therefore, the capacity for negative maternofetal effects and high altitude pathologies remains, and potential maladaptations to high altitude—distinct in each population—warrant consideration.

### 8.1 Indigenous highlanders

Birth mandates increased blood flow through the newborn lungs. This response is opposed by high altitude due to alveolar hypoxia (Dempsey & Morgan, 2015). Therefore, while previously viewed in terms of its benefit to diffusion capacity, the pulmonary remodelling of the highland native may be compromised, resulting in pulmonary hypertension—a phenomenon exacerbated by erythrocytosis and accompanying increases in blood viscosity (Dempsey & Morgan, 2015). Reversal of pulmonary hypertension is observed following prolonged sea level residence (Penaloza & Arias‐Stella, 2007), and increased production of nitric oxide in the lungs of those native to high altitude may help maintain flow through the pulmonary vasculature (Beall et al., 2001). Another consideration is chronic mountain sickness, an incapacitating syndrome occurring in lifelong altitude residents characterized by excessive erythrocytosis, fatigue and cyanosis, and in some cases accompanied by pulmonary hypertension and heart failure (León‐Velarde et al., 2005). Approximately 10% of Andean adults experience chronic mountain sickness. This number is comparable to that observed in Coloradan newcomers and far greater than the 1% of Tibetan highlanders that suffer from the condition (Moore, 2017); preliminary data suggest that chronic mountain sickness is absent in those from the Ethiopian highlands (Getu & others, 2022). Finally, hypoxia‐induced postnatal remodelling of the pulmonary vasculature may also be linked with right ventricular hypertrophy (Penaloza & Arias‐Stella, 2007).

### 8.2 Highland newcomers

Exposure to hypoxia may induce a remodelling of the pulmonary vasculature, particularly by increasing the amount of smooth muscle cells in the distal pulmonary arterioles. This additional muscularization elevates vascular resistance and contributes to rates of pulmonary hypertension that are greater in highland‐born adolescents compared to lowland‐born (Sime et al., 1963). Compared to indigenous highlanders of the Himalayas, newcomers born and raised in the Rocky Mountains exhibit higher pulmonary arterial pressures and pulmonary vascular resistance to hypoxia (Groves et al., 1993; Penaloza et al., 2008). Lower levels of nitric oxide were reported in some (Ge et al., 2011) but not all (Weitz et al., 2013) investigations comparing newcomers to indigenous highlanders and may contribute toward a greater observed prevalence of chronic mountain sickness in newcomers compared to native highlanders (Wu, 2005). The evidence for pulmonary maladaptation in highland newcomers is further supported by data demonstrating a relatively greater degree of re‐entry high altitude pulmonary oedema (which occurs upon returning to high altitude following short stints at lower elevations) in children of Han Chinese descent compared to natives of the Tibetan plateau (Penaloza et al., 2008). Finally, data collected on over 30,000 children throughout the highlands (1800 m) and lowlands (450 m) of Austria found an association between atopic asthma and altitude of residence where the risk of asthma hospitalization increased 7% for every 100 m increase in altitude (Kiechl‐Kohlendorfer et al., 2007).

### 8.3 Highland sojourners

Though spared the potential insult of perinatal exposure to high altitude, the adult sojourner to high altitude remains susceptible to maladjustments elicited by the low oxygen environment. Pulmonary arterioles constrict when exposed to hypoxia elevating pulmonary arterial pressure and contributing toward an added strain on the right ventricle (Dempsey & Morgan, 2015). Upon return to sea level, pulmonary hypertension can persist in the highland sojourner for up to a few days (Anand et al., 1990; Dorrington et al., 1997), but hypoxia‐induced remodelling of the pulmonary vasculature is reversible within 1 week (Hilty et al., 2016). Even acute exposure to high altitude bears with it risks of acute mountain sickness (often benign and self‐resolving given acclimatization or return to sea level), as well as high altitude cerebral and pulmonary oedema (Mallet et al., 2023). Recently, the Global REACH expedition to Cerro de Pasco, Peru (∼4300 m) produced a robust battery of information regarding highland sojourners and found, for example, that ascent to high altitude evokes an inflammatory response, heightened adrenergic activity and persistent respiratory alkalosis, each of which could influence exercise performance if temporarily retained upon return to sea level (Brewster et al., 2023; Hansen et al., 2022; Steele et al., 2022).

### 8.4 Summary of maladaptation

According to information readily available online, 67% of medalists in the men's and women's marathons in the 2020 Olympics, and perhaps 100% of medalists in the 2020 men's and women's Olympic 5000 m track event, were either highland natives or highland newcomers. Numbers like these can lead us to assume that being born and raised in a high altitude setting necessarily confers an ergogenic advantage. However, by limiting our scope to Olympic‐level athletes, we preselect against those whose physiology was hindered by highland birth. Although examining the physiology of Olympians illuminates the potential benefit of environmental cues, it does little to teach about the detriment of these same environmental insults. After all, in the 4‐year Olympic cycle from 2016 to 2020, the high altitude of Kenya may have helped produce a few hundred Olympians. But that same 4‐year span witnessed the birth of over 50,000 children in the highlands of Eldoret, Kenya (elevation 2090 m, considered a hub for Kenyan endurance prowess) and if perinatal trends are transferable from the Denver highlands (Bailey et al., 2019) to Eldoret, nearly 6000 of those births were small for their gestational age (Statista, 2019). In this case, what is *un*seen is perhaps as relevant as what is seen, and while 12% of children born at high altitude risk the lifelong physiological impairments accompanying low birth weight (Bailey et al., 2019), a comparatively minuscule number of children born at high altitude become elite athletes. Yet, given the already‐improbable prospect of Olympic glory, even an infinitesimal edge toward this end may prove significant. And for those hopeful parents longing for their offspring to achieve Olympic glory, like Icarus ascending towards the sun, the potential enhancement in athletic prowess conferred by a highland birth may prove a lustre too tempting to forgo.

## 9 FURTHER CONSIDERATIONS

### 9.1 Variation in ventilatory characteristics amongst indigenous highlanders

Just as geographical and historical differences exist between sojourners, newcomers and indigenous highlanders, and contribute towards the anatomical and physiological distinctions between these groups, so too geographical and historical differences exist *within* these populations. For example, the Tibetan plateau, covering approximately one million square miles, is larger than the Andean altiplano, and whereas the indigenous highlanders of the Andes have occupied their high altitude setting for 14,000 years, Tibetans have inhabited their high altitude environment for perhaps as long as 30,000 years (Beall, 2013; Moore, 2021). These distinctions in time and place are exemplary of additional distinctions in anatomy and physiology within these indigenous groups (Beall, 2014; Niclou et al., 2023).

Based on early experimental results, especially on Andean natives (Brutsaert et al., 2005), preliminary views posited that, compared to acclimatized sojourners, highland birth lowers resting ventilation as well as chemosensitivity as assessed by the hypoxic ventilatory response (HVR). However, subsequent research on Tibetan highlanders challenged these conclusions, showing that Tibetans had resting ventilations and HVRs that were similar to, or greater than, acclimatized sojourners (Zhuang et al., 1993). Therefore, contemporary thought states that Andean highlanders exhibit a desensitization of the carotid bodies characterized by a lower resting ventilation and lower HVR compared to Tibetan highlanders (Wu & Kayser, 2006; Zhuang et al., 1993). In addition to the distinctions in ventilation and HVR, $A-aD_{O_{2}}$ appears to be similarly narrowed amongst indigenous highlanders (Moore et al., 1998), though resting $S_{aO_{2}}$ may be better preserved in the Andean than the Tibetan native (Beall, 2007). Some insights exist regarding exercising $S_{aO_{2}}$ (Brutsaert, 2007) but a greater battery of research would permit stronger comparisons between highland groups.

**FIGURE 5:**
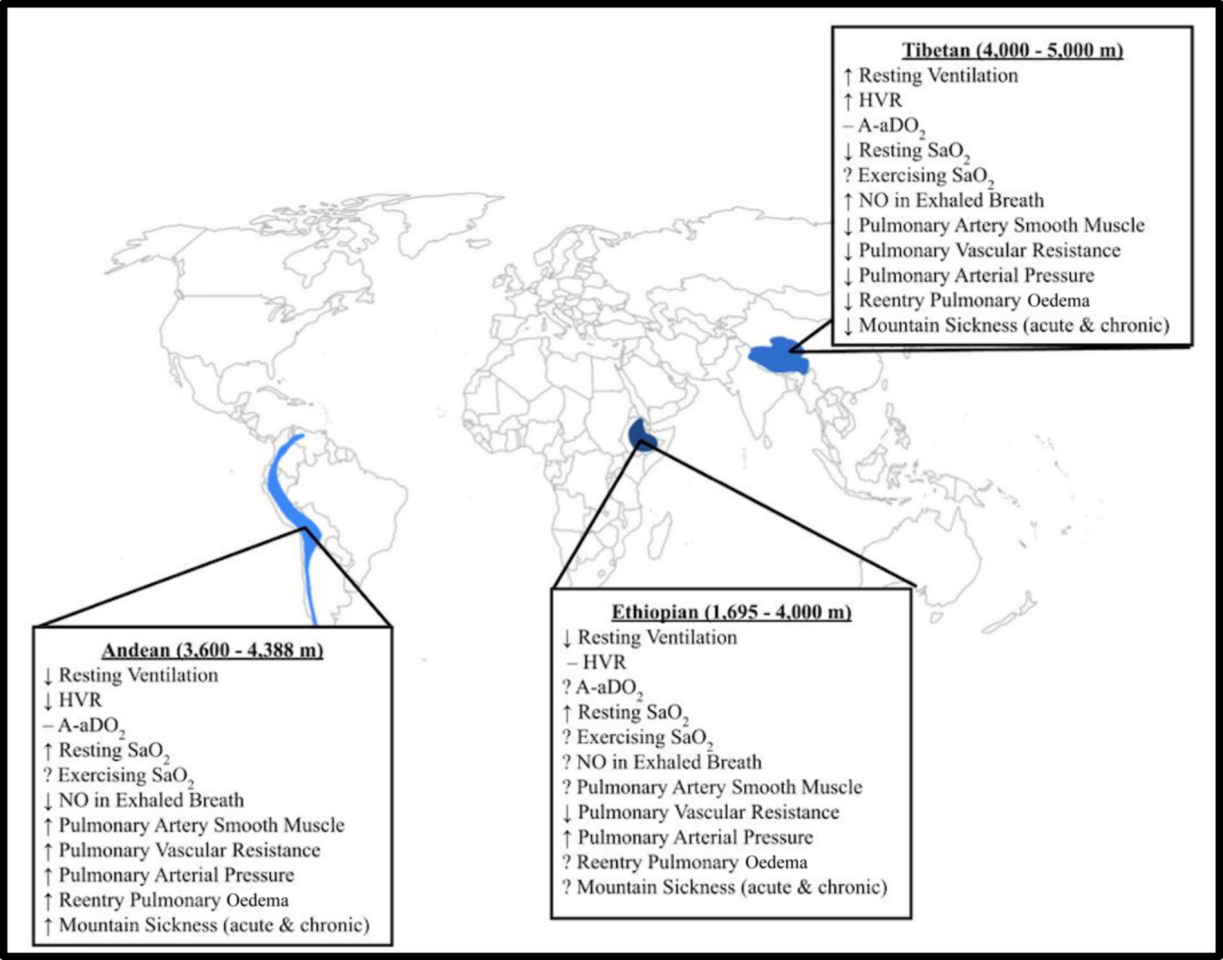
Distinct pulmonary adaptations among the three geographic regions where humans have adapted to high altitude: Andean, Ethiopian (Semien) and Tibetan plateaus. Map compares Andean versus Tibetan versus Ethiopian indigenous highlanders; adapted from Bigham (2016) as well as Beall (2014) and Niclou (2023). $A-aD_{O_{2}}$, alveolar‐arterial $P_{O_{2}}$ difference; HVR, hypoxic ventilatory response.

Therefore, in questioning specifically whether indigenous highlanders experience an ergogenic edge for sea‐level performance, differentiating between indigenous populations proves necessary as ancestral and geographical variations point towards distinct adaptations (Figure 5). These distinct responses influence the frequency and severity of pulmonary adaptations, be they beneficial or detrimental to health and human performance. Although less is known regarding Ethiopian adaptations, Andean versus Tibetan comparisons reveal that the Tibetans are characterized by greater resting alveolar ventilation and chemosensitivity (Brutsaert, 2007; Moore et al., 1998). Although the relatively low ventilation of the Andean does not appear to be deleterious to health, nor the relatively high HVR of the Tibetan advantageous (Beall et al., 1997), the Tibetan pulmonary profile may contribute towards lower rates of mountain sickness. A number of genomic underpinnings for altitude adaptation have been elucidated and some genetic distinctions between highland groups have been identified (Bigham, 2016). Investigations continue to pinpoint the aetiological differences between indigenous populations, which likely relate to various degrees of genetic admixture and gene–environment interactions (Brutsaert, 2007; Moore et al., 1998). Of relevance to the discussion on Olympic aptitude, many of the noted distinctions between Andeans and Tibetans have been observed at rest, and some preliminary data indicate that during exercise differences deteriorate (Harman et al., 2023).

### 9.2 Potential sex differences in response to pulmonary adaptations with high altitude exposure

Women may be more limited by pulmonary constraints when performing maximal aerobic exercise compared to men due to their smaller conductive airways and lung sizes (even when matched for height) (Dominelli et al., 2018; Sheel et al., 2009). This potential limitation is reflected in the relatively greater susceptibility of women to developing exercise‐induced arterial hypoxaemia (Dominelli & Sheel, 2019). Further evidence suggests there may be sex differences in the adaptive responses of the pulmonary system to high altitude for sojourners (for further discussion see a recent review on this topic: Raberin et al., 2024). Accordingly, it is plausible that the exercise performance of women may benefit differentially from pulmonary adaptations conferred with high‐altitude birth. As with elite men, anecdotal evidence supports the possibility that highland birth benefits sea‐level performance in women, as might be the case for the Ethiopian‐born Dutch athlete Sifan Hassan, who achieved a historic treble in the recent Paris Olympics with bronze at 5000 m, bronze at 10,000 m, and gold in the women's Olympic marathon. Hassan was born in the Munessa District of Oromia, Ethiopia, where the altitude ranges from 1500 m to over 4100 m. Direct comparisons of the adaptations of highland men and women are lacking, however, with few studies including women in their analyses. Ultimately, future work is needed to directly investigate sex differences in the pulmonary adaptations of highland sojourners, highland newcomers and indigenous highlanders.

## 10 SUMMARY

The physiological remodelling incurred by the indigenous highlander (e.g., those native to the Andean mountains or Tibetan plateau) is distinct from the highland newcomer (e.g., Han migrants to Tibet or those from Leadville, Colorado). The responses of both, however, may prove beneficial in environments where oxygen is a limiting factor—be it the high altitude setting or endurance performance at sea level when approaching the limits of oxygen delivery. In addition to inheritable adaptations, evidence suggests that being born at high altitude initiates developmental perturbations of the lungs which promote tissue oxygenation, and that this differs from patterns observed during adult acclimatization. Given a healthy pregnancy—which is not assured as perinatal hypoxia risks several maladaptations—these perinatal responses may confer an athletic advantage during competitions at sea level. Contrary to this hypothesis, data on elite Kenyan runners at 1500 m observed a pulmonary system comparable to elite runners elsewhere (Foster et al., 2014). Further evidence is therefore warranted, directly comparing the pulmonary adaptations and sea‐level endurance performance of indigenous highlanders versus lowlanders.

In this review we posed the question of whether highland born athletes traveling to sea level for endurance exercise competitions experience an ergogenic advantage due to pulmonary adaptations accompanying their highland birth. In addition to the degree of generational high altitude exposure, three prominent aspects need also be considered when translating prior research to this present inquiry: (1) *athletes*—comparisons between mountaineering Tibetans and sedentary lowlanders must be tempered in light of differences in training status; (2) *sea level*—assumptions that what is true at high altitude (e.g., alterations in $S_{aO_{2}}$) remains consistent at sea level require verification; and (3) *exercise*—even if studies take highland born athletes, transport them to sea level, and compare them with lowland born athletes, data obtained in the resting state (e.g., differences in resting ventilation, hypoxic ventilatory response, etc.) may not correspond to that which is experienced during maximal exercise. Additionally, the current discussion considered the ramifications of highland birth for sea level performance exclusively from a perspective of changes to the structure, function and control of the lungs. The highland environment influences more than pulmonary physiology, however, and is perhaps most well‐known for influencing the haematological profile. Keeping in mind the physiological crosstalk between tissues, as well as the capacity for perinatal hypoxia to influence anatomy and physiology beyond the lungs alone, any ergogenic potential from highland birth may result from adaptations elsewhere in the oxygen cascade. For example, consistent with the idea that indigenous highlanders outperform acclimatized lowlanders at high altitude (Brutsaert, 2008), evidence suggests that those native to high altitude have greater cardiac outputs, likely due to larger stroke volumes (Moore et al., 1998). Furthermore, several groups have noted better sea level running economy (oxygen cost of running at submaximal paces) in athletes born at high altitude (Mooses et al., 2015; Saltin, Larsen et al., 1995), perhaps due to differences in factors related to the skeletal muscle (Saltin, Kim et al., 1995; Scott et al., 2009) or anatomical structure of some athletes (Larsen et al., 2004; Mooses et al., 2015). Although a wider perspective such as this was beyond the scope of the present paper, a more comprehensive review is likely warranted. This subsequent review would encompass an integrative physiological perspective on this topic, thereby extending the current discussion beyond pulmonary adaptations alone. Finally, for the sake of the present review, and in line with previous literature, indigenous highlanders were defined as multigenerational populations born, raised and living above 2500 m. But many of the contemporary cradles of human endurance capacity (e.g., Iten, Kenya and Addis Ababa, Ethiopia) rest slightly below this threshold. The degree to which a 500 m descent changes the present discussion (i.e., perinatal adaptations at 2000 m compared to 2500 m) is an area for future research.

Ultimately, a cursory consideration of the potential ergogenic advantages imbued by a highland birth may lead one to conclude that had Jim Ryun been born in Mexico City he would have captured gold in 1968. Keeping in mind the possibility for pulmonary hypertension in the hypoxic environment, however, the risk of maladaptations must also be considered. Had Jim Ryun been born in Mexico City, perhaps he never would have been in the race at all. But if one can circumvent altitude‐related maladaptations (perhaps through heritable characteristics), developmental alterations accompanying highland birth—particularly a greater ability to diffuse oxygen during exercise—may present benefits for endurance competitions at sea level.

## AUTHOR CONTRIBUTIONS

All authors have read and approved the manuscript. Conceptualization: Marissa N. Baranauskas, Keren Constantini, Hunter L. Paris, Ren‐Jay Shei, Chad C. Wiggins, Cooker Perkins Storm. Writing: Hunter L. Paris, Marissa N. Baranauskas, Keren Constantini, Ren‐Jay Shei, Peyton E. Allen, John R. Jadovitz, Chad C. Wiggins, Cooker Perkins Storm. Review & editing: Hunter L. Paris, Marissa N. Baranauskas, Keren Constantini, Ren‐Jay Shei, Peyton E. Allen, John R. Jadovitz, Chad C. Wiggins, Cooker Perkins Storm. Visualization: Peyton E. Allen, Cooker Perkins Storm, Chad C. Wiggins, Hunter L. Paris. All authors have read and approved the final version of this manuscript and agree to be accountable for all aspects of the work in ensuring that questions related to the accuracy or integrity of any part of the work are appropriately investigated and resolved. All persons designated as authors qualify for authorship, and all those who qualify for authorship are listed.

## CONFLICT OF INTEREST

R‐J.S. is an employee as a Medical Science Liaison of a pharmaceutical company focused on Rare Disease. The views herein are the author's (R‐J.S.) personal views and unrelated to his job duties, do not constitute endorsement by his employer, nor do they represent the views of his employer. The authors have no other conflicts of interest to declare.

## FUNDING INFORMATION

No funding was received for this work.
